# A single dose of Ultraviolet-A induces proteome remodeling and senescence in primary human keratinocytes

**DOI:** 10.1038/s41598-021-02658-5

**Published:** 2021-12-02

**Authors:** Hellen Paula Valerio, Felipe Gustavo Ravagnani, Graziella Eliza Ronsein, Paolo Di Mascio

**Affiliations:** Department of Biochemistry, Institute of Chemistry, University of São Paulo, São Paulo, 05508-000 Brazil

**Keywords:** Cell biology, Biochemistry, Proteomics

## Abstract

Epidermal photoaging contributes to skin fragility over time and it is a risk factor for skin cancer. Photoaging has been associated for a long time with exposure to Ultraviolet-A (UVA) light, the predominant component of the solar ultraviolet radiation. While the cellular mechanisms underlying UVA-induced photoaging in the dermis have been well characterized, UVA’s action on the epidermis remains elusive. Here, proteomic analysis was conducted to derive the cellular responses induced by an environmentally relevant dose of UVA in primary human keratinocytes. We also investigated the effects of UVA on non-transformed immortalized keratinocytes (HaCaT cells), bearing potentially oncogenic mutations. We showed that UVA induces proteome remodeling and senescence in primary keratinocytes, eliciting potent antioxidant and pro-inflammatory responses. Additionally, we showed that UVA modulates the secretory phenotype of these cells to the extent of inducing paracrine oxidative stress and immune system activation in pre-malignant keratinocytes. These observations offer insights into the cellular mechanisms by which UVA drives photoaging in the skin.

## Introduction

The solar ultraviolet radiation that reaches the Earth comprises about 5% of UVB (290–320 nm) and 95% of UVA light (320–400 nm), and both are strongly associated with skin photoaging and tumorigenesis^1^. While the mechanisms underlying UVB carcinogenicity have been well-described for at least 60 years^2^, involving direct generation of bipyrimidine photoproducts, UVA has only been classified as “probably carcinogenic to humans” by the World Health Organization’s International Agency for Research on Cancer in 2009^1^ and many of its cellular effects remain unknown. This is due to the fact that UVA is poorly absorbed by canonical nucleotides, causing much less direct DNA damage than UVB^3^. However, UVA can still be absorbed by other cellular chromophores, generating photoexcited species as singlet oxygen, as well as free radicals, and consequently causing oxidative damage in cells^4^.

Ultraviolet light drives photoaging both at the level of the dermis and epidermis. UVA’s effect on the dermis have been widely studied, likely because UVA is more efficient in reaching this skin layer than UVB^5^. Dermal changes during aging reflect senescence as well as death of fibroblasts, which are constantly remodeling the connective tissue by secretion of soluble factors and extracellular matrix repertoire, both under homeostasis and cellular stress conditions^6^. In this sense, UVA have been shown to induce alterations in the gene expression signatures of fibroblasts, modulating their secretory capacity, and eventually leading to apoptosis and senescence^5,7^. Similarly, aging also impacts ultrastructure and function of the epidermis, interfering with skin thickness, barrier capacity, prevention of water loss, hydration maintenance and reepithelization post wound healing^8^. Epidermis balance is dependent on the proliferative and differentiation capacities of keratinocytes, which are the main cell type of this skin layer and are in constant renewal. While UVB’s action on the epidermis is well-characterized^9^, UVA-induced cellular mechanisms underlying epidermal alterations during aging are not as well understood. Lethal UVA doses have been shown to induce apoptosis in epidermal keratinocytes^10^ and melanocytes^11^, but to date the impact of low doses of UVA on epidermal photoaging has not been well characterized.

Here, proteomic analysis was conducted to derive the phenotypic signature induced by a low dose of the UVA component of the sunlight in primary human keratinocytes. Our results indicate that, upon exposure to UVA radiation, keratinocytes may enter senescence and elicit paracrine responses in neighboring initiated cells. We also investigated the effects of UVA on immortalized human keratinocytes that harbor potentially oncogenic mutations and dysfunctional components of the senescence machinery, providing a proteomic characterization of the differential sensitivity of the two cell types to the radiation.

## Results

### A single, low dose of UVA light induces extensive proteome remodeling in primary keratinocytes

**Figure 1:**
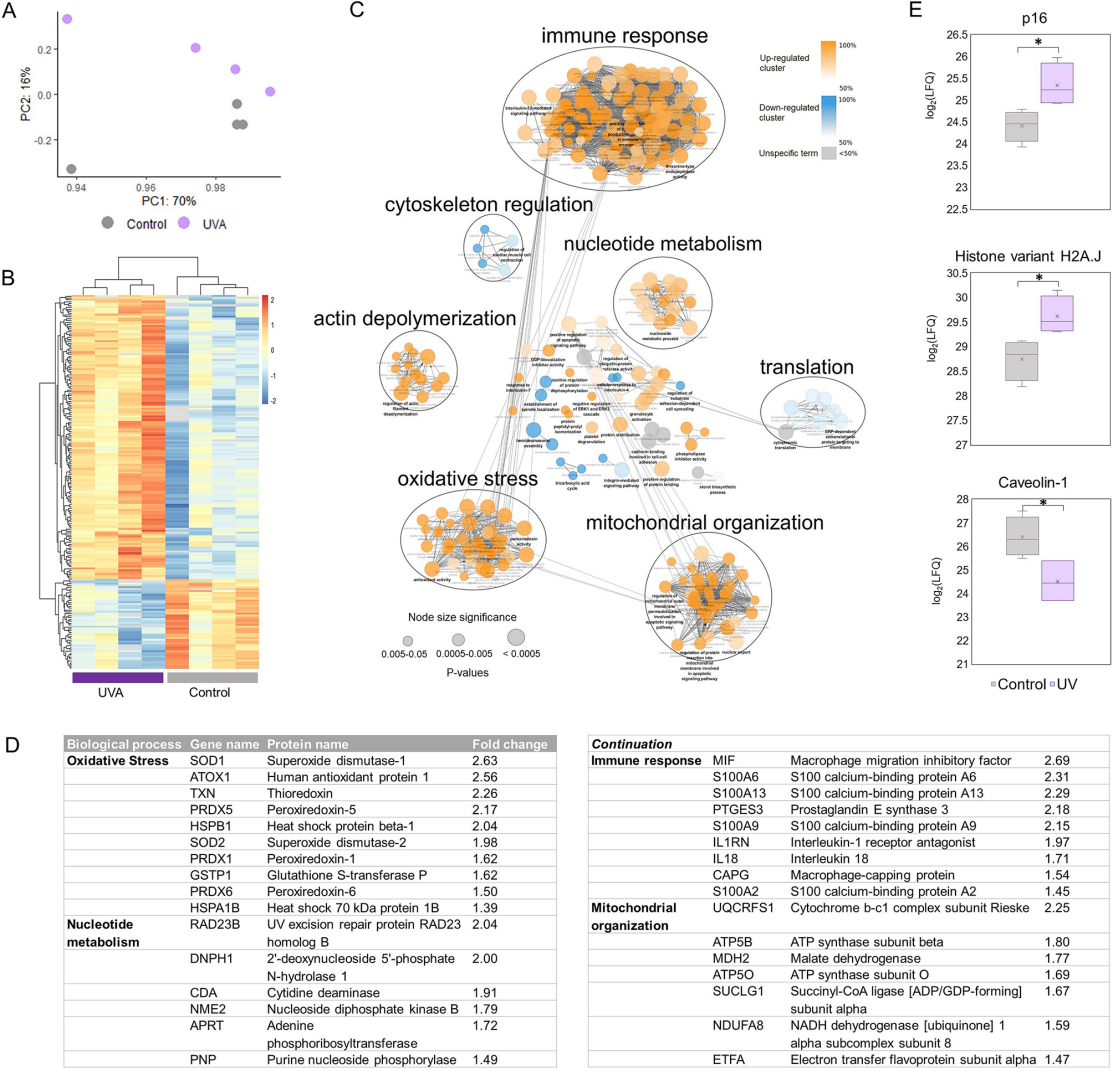
Proteome remodeling induced by UVA light in primary human keratinocytes. (**A**) Principal component analysis of UVA-irradiated and control samples obtained by analyzing NHEK cells proteome 24 h after exposure to the radiation (n = 4 per group). (**B**) Hierarchical clustering of differentially regulated proteins (two-tailed Student’s *t*-test, 0.05 FDR correction, with S_0_ parameter value of 0.1) in NHEK cells 24 h after exposure to UVA light. The color gradient represents z-scored Label Free Quantification (LFQ) intensities and columns represent replicates. (**C**) Enrichment network of the up and down-regulated clusters identified in the heatmap. Enriched GO-terms for biological processes are shown as nodes, interconnected according to the number of genes shared between them. The size of the nodes reflects the significance of the terms (*p*-value < 0.05, right-sided hypergeometric test, Benjamin-Hochberg FDR correction). Semantically-related biological processes were clustered and labeled with AutoAnnotate. (**D**) Most significantly up-regulated proteins in NHEK cells 24 h after exposure to UVA light. (**E**) Boxplots of the log_2_ LFQ intensities of p16, caveolin-1 and histone H2A.J in irradiated and control NHEK cells 24 h after treatment. Asterisks indicate significant differences (*corrected *p*-value < 0.05) determined by Student’s *t*-test post FDR correction. The x inside the box represents the mean of all values.

We first tested if exposure to an environmentally relevant and non-cytotoxic low dose of UVA light could promote changes in protein levels of normal human epidermal keratinocytes (NHEK cells). For that purpose, we irradiated NHEK cells with a dose of 6 J/cm^2^ of UVA light using a solar UVA spectrum simulator, and monitored changes in protein levels by shotgun proteomics. Importantly, cell viability was above 90% with this UVA dose. Proteins were extracted from irradiated and mock-treated cells 24 h after irradiation, digested into peptides and analyzed by mass spectrometry. Protein groups were considered differentially expressed if, after hypothesis testing (Student’s T-test), the FDR-corrected *p*-values were lower than 0.05. The label-free quantification and results of the statistical test associated with this dataset are presented in the first tab of Supplementary Spreadsheet S1. Principal component analysis of the variables shows a radiation-dependent clusterization effect (Fig. 1A). Among the 2807 identified proteins, 253 were differentially regulated 24 h after UVA irradiation, comprising a cluster of 205 up-regulated proteins and a cluster of 48 down-regulated proteins (Fig. 1B).

To evaluate systematically the biological meanings associated with these changes, significantly up and down-regulated proteins were used to generate an enrichment network based on Gene Ontology’s terms through ClueGO^12^ (Fig. 1C). The complete list of enriched biological processes is available in the first tab of Supplementary Spreadsheet S2. Additionally, semantically related GO-terms were clustered and labeled with AutoAnnotate to facilitate visualization of the network. The ClueGO analysis revealed that the main biological processes associated with up-regulated proteins in NHEK cells are oxidative stress, immune response, nucleotide metabolism and mitochondrial metabolism, whereas down-regulated proteins are enriched for translation, cytoskeleton regulation, and cell adhesion (see Fig. 1D for a table containing key up-regulated proteins and significantly enriched biological processes).

Differential regulation of three markers of senescence (the tumor suppressor p16, histone H2A.J and caveolin-1)^13–15^ (Fig. 1E), together with exacerbated pro-inflammatory and antioxidant responses (Fig. 1C), led us to wonder if a single, low dose of UVA could impact cellular proliferation and induce cellular senescence. In addition, senescent cells usually display an increased number of mitochondria with decreased membrane potential^16^ accompanied by a reduction in mitophagy^17^, leading to the release of mitochondrial proteins and intensified ROS production^15^. Mitochondrial damage also plays an important role in UVA’s biological action^18^. The up-regulation of several structural mitochondrial proteins in irradiated NHEK cells, such as components of the respiratory chain, seems consistent with the increase in mitochondrial mass usually observed in senescent phenotypes^17^.

Furthermore, analysis of the differentially expressed proteins also shows that proteostasis is importantly affected by UVA light. It has been observed that accumulation of unfolded proteins during stress-induced senescence^19^ may lead to the activation of the unfolded protein response in the endoplasmic reticulum, followed by a reduction in protein synthesis^20^. However, protein synthesis is required for production of the senescence-associated secretory phenotype (SASP) and, as a consequence, senescent cells usually suffer from proteotoxic stress^21^. Accordingly, we found down-regulation of ribosomal subunits and translation regulators, and up-regulation of several heat-shock proteins, transcription factors, translation regulators and proteasome subunits in irradiated cells.

### UVA light induces a senescent phenotype in primary keratinocytes

**Figure 2:**
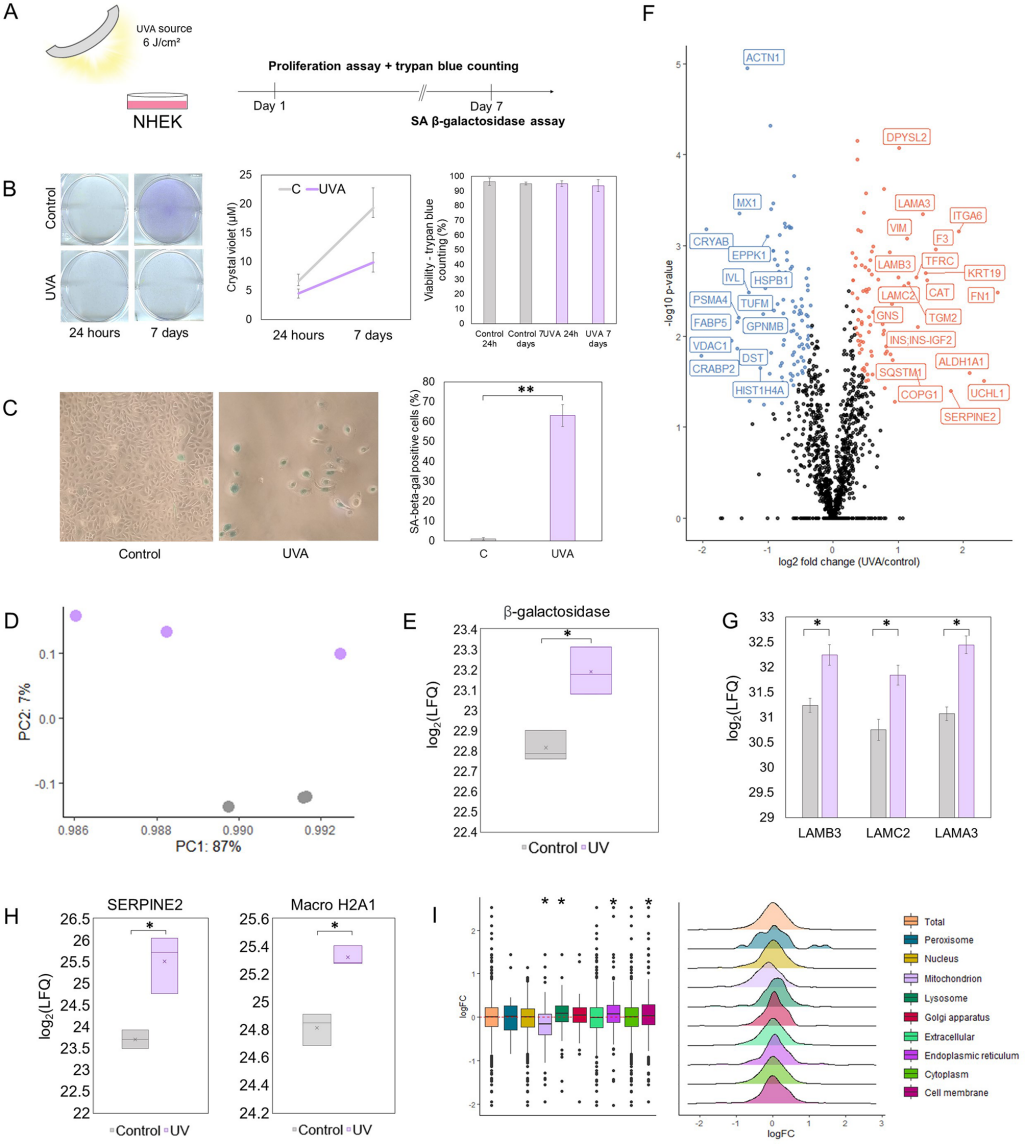
Long-term implications of UVA-induced cellular senescence. (**A**) Simplified workflow of the proliferation and β-galactosidase assays used to confirm senescence induction by UVA light in primary keratinocytes. (**B**) NHEK cells were exposed to UVA light or mock-treated and stained with crystal violet after 24 h and 7 days. Representative images of the stained plates are shown in the left and growth curves are shown in the right, with points representing means, and error bars representing standard deviation values (n = 3). Trypan counting data is shown in the barplot, with bars representing means and error bars representing standard deviation (n = 3). (**C**) NHEK cells were stained for senescence-associated β-galactosidase 7 days after exposure. Representative images of control and UVA-irradiated cells are shown next to a barplot representing the means of the percentage of stained cells in each condition as well as standard deviations. Asterisks indicate significant differences (**p-value < 0. 0.001) determined by Student's t-test. (**D**) Principal component analysis of UVA-irradiated and control samples obtained by analyzing NHEK proteome 7 days after exposure to UVA radiation (n = 3 per group). (**E**) Boxplot of the log_2_ LFQ intensities of beta-galactosidase in irradiated and control NHEK cells 7 days after irradiation. The x represents the mean of all values. Asterisks indicate significant differences (*corrected *p*-value < 0.05) determined by Student's t-test post FDR correction. (**F**) Proteomics analysis of NHEK cells exposed or not to UVA light (cells were lysed 7 days post-treatment). Significantly up and down-regulated proteins are shown in the volcano plot, highlighted in orange and blue, respectively. Significance was defined in this plot by a 0.05 FDR and labels were shown for proteins with log_2_(Fold Change) > 0.5 or < 0.5 (n = 3). (**G**) Differential levels of laminin subunits (*LAMB3*, *LAMC2*, *LAMA3*) in irradiated and control samples 7 days post-exposure. Bars represent mean log_2_(LFQ) values as measured by mass spectrometry and error bars represent standard deviation. Asterisks indicate significant differences (*corrected *p*-value < 0.05) determined by Student's t-test post FDR correction. (**H**) Boxplot of the log_2_ LFQ intensities of *SERPINE2* and core histone macro-H2A.1 in irradiated and control NHEK cells 7 days after irradiation. The x represents the mean of all values. Asterisks indicate significant differences (*corrected *p*-value < 0.05) determined by Student's t-test post FDR correction. (**I**) Compartment-specific proteomic analysis^27^ of the log_2_ fold changes of irradiated NHEK cells in relation to controls. Proteins were assigned to compartments according to GO-terms and each compartment was tested for difference against the whole proteome (Wilcoxon rank sum test with 0.5% FDR correction).

The up-regulation of immune and stress responses in UVA-exposed keratinocytes, together with differential organization of the mitochondrial proteome and altered proteostasis are coherent with some of the metabolic changes that have been previously described in senescence, such as increased mitochondrial mass and proteotoxic stress^15^. To confirm that UVA was inducing a senescent phenotype in primary keratinocytes, we used two well-characterized senescence assays (Fig. 2A). First, we employed the crystal violet assay to assess cellular proliferation^22^. Cells were also counted by the trypan blue exclusion method to assure that crystal violet staining reflected growth rates and not viability loss. Our results showed that exposure to UVA light impacts cellular growth overtime until at least 7 days after exposure, without concomitant increases in membrane permeabilization to trypan blue (Fig. 2B). Second, cells were tested for one of the main hallmarks of senescence, the senescence-associated (SA) β-galactosidase^23^. Irradiated NHEK cells showed an increased accumulation of this protein in relation to controls 7 days after exposure to the radiation, besides also exhibiting morphological changes (a large and flat appearance, Fig. 2C).

The results obtained with the senescence assays lead us to explore the proteomic profiling of irradiated NHEK cells 7 days after exposure to UVA (the label-free quantification of this dataset and results of the statistical test are presented in the second tab of Supplementary Spreadsheet S1). Besides promoting a deep remodeling of the proteome after 24 h of exposure, UVA radiation produced long-lasting effects on the proteome of primary keratinocytes, leading to clusterization of the variables in a treatment-dependent fashion in the PCA (Fig. 2D). Importantly, our proteomic experiments showed an increase in β-galactosidase levels 7 days after UVA exposure in irradiated cells compared to controls (Fig. 2E), confirming the results obtained with the staining assay of SA β-galactosidase. By inspecting proteins with larger differences between UVA-treated and control cells (Fig. 2F), we found senescence-like changes in the proteome of UVA-exposed cells. Notably, irradiated cells have increased levels of laminin subunits (*LAMA3, LAMB3, LAMC*) and *SERPINE2*, proteins usually secreted in SASP^24,25^ (Fig. 2G,H). Also, UVA-exposed cells have accumulation of core histone macro-H2A.1 (Fig. 2H), a key player in regulation of SASP gene expression^26^, and a persistent, long-lasting up-regulation of redox-responsive proteins, such as catalase.

Additionally, increased levels of sequestosome-1 (*SQSTM1*) were observed in irradiated cells (Fig. 2F), suggesting late activation of autophagic removal after light stress. In fact, by analyzing compartment-specific changes in the proteome according to the methodology developed by^27^, we found a significant reduction in the fold changes of mitochondrial proteins in relation to the whole proteome in the long-term, as well as an increase in the fold changes of the lysosomal proteins (Wilcoxon test, 5% FDR correction) (Fig. 2I). In this regard, there are evidences that SASP production by senescent cells elicit a proteotoxic response, affecting cellular metabolism and triggering autophagy as a mechanism for coping with protein unfolding^28^. UVA has also been described to impair autophagic flux, which could explain the late cellular repair response^29^.

### UVA light induces distinct phenotypic signatures in primary and immortalized non-tumorigenic human keratinocytes

**Figure 3:**
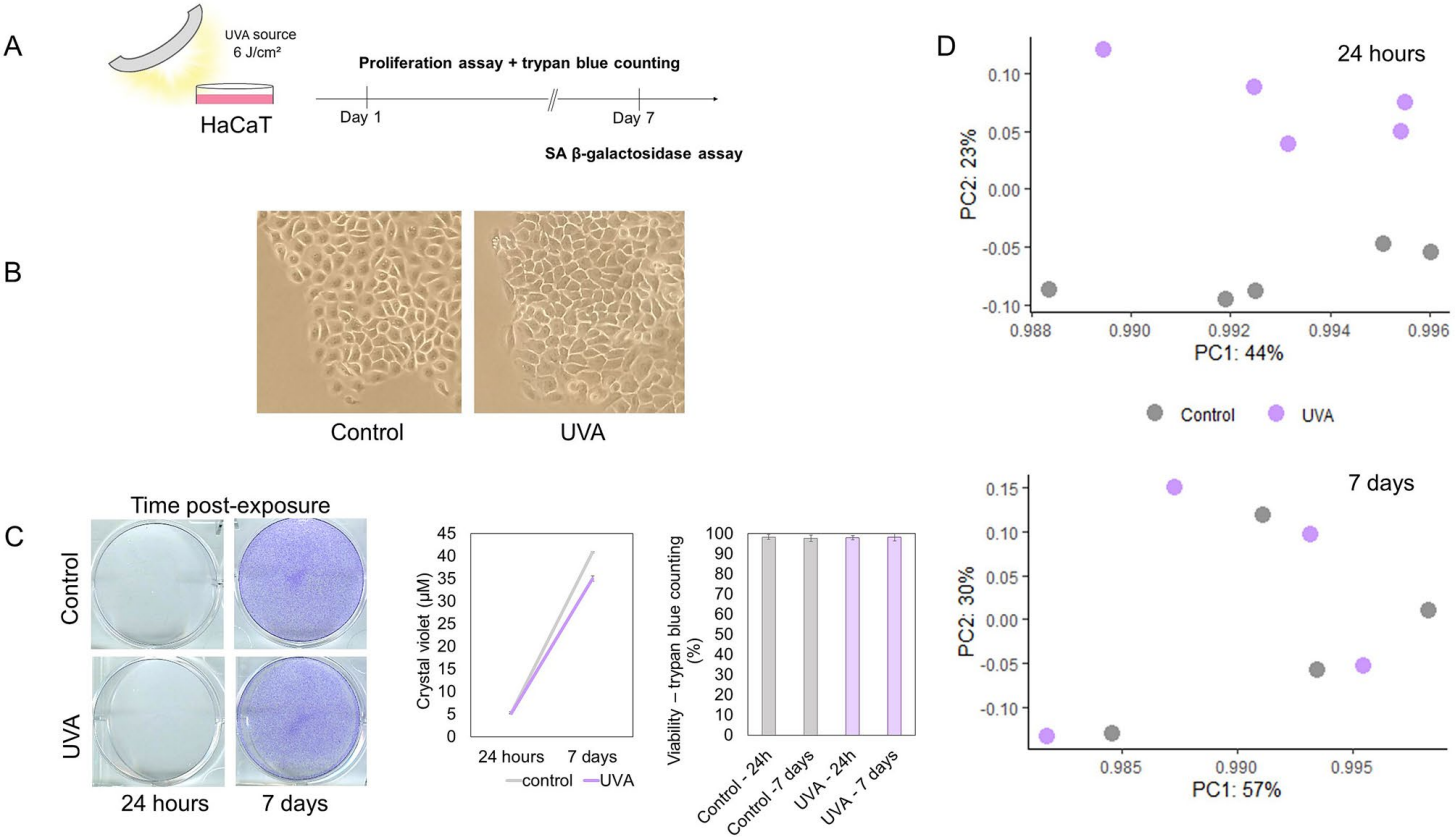
Immortalized HaCaT cells are resilient to UVA light. (**A**) Simplified workflow of the assays used to confirm senescence resistance in immortalized HaCaT cells exposed to UVA light. We acknowledge Servier Medical Art (https://smart.servier.com) for providing the illustrations. (**B**) Representative images of control and UVA-irradiated HaCaT cells after SA β-gal staining (n = 3 independent experiments). The assay was performed 7 days after irradiation and no staining was observed in any condition. (**C**) HaCaT cells were exposed to UVA light or mock-treated and stained with crystal violet after 24 h and 7 days. Representative images of the stained plates are shown in the left, and growth curves are shown in the right, with points representing means and error bars representing standard deviation values (n = 3 independent experiments). Trypan counting data are shown in the bar plot, with bars representing means and error bars representing standard deviation (n = 3 independent experiments). (**D**) Principal component analysis of UVA-irradiated and control samples obtained by analyzing the proteome of HaCaT cells 24 h after irradiation (n = 5 per group) as well as 7 days after exposure to the radiation (n = 4 per group).

We were also interested in how UVA light affects keratinocytes at very early stages of tumorigenesis. With that in mind, we used HaCaT cells as a model to understand the effects of UVA light in pre-malignant keratinocytes (Fig. 3A). HaCaT cells do not harbor any viral sequences^30,31^, and were likely immortalized as a consequence of UV-like mutations in p53^32^, similar to the ones found in skin carcinomas and pre-malignant lesions^33^. Therefore, this lineage is used as a model to study very early stages of skin tumorigenesis^34^. Besides inactivation of the two alleles of p53, HaCaT presents other characteristics of an initiated cell line, such as increased telomerase activity, silencing of p16, and defective regulation of p21 expression and function^35^, all key components of the senescence machinery. HaCaT cells are also able to undergo tumorigenic conversion by overexpression of a single proto-oncogene (NF-κB)^35^. Despite these alterations, HaCaT cells have a stable chromosome content and remain non-tumorigenic^30^.

HaCaT cells show a distinct pattern of proteome remodeling in response to UVA-induced stress when compared to NHEK cells. As expected, HaCaT cells do not show up-regulation of classical senescence markers, as p16, or of antioxidant and pro-inflammatory enzymes, and do not stain positive for the accumulation of SA β-galactosidase under exposure to UVA light in relation to controls (Fig. 3A,B). Moreover, while NHEK cells exhibit a pronounced difference in growth rates between groups, HaCaT cells present only a mild proliferation reduction when irradiated, recovering their proliferative capacity in the long-term (Fig. 3C). A proteomic screening of UVA-irradiated and control HaCaT cells in the short and long-terms (24 h and 7 days after irradiation, respectively) reveals that in the short-term UVA impacts the proteome composition, but in the long-term the proteome returns to homeostasis (Fig. 3D). The label-free quantification of these datasets and the results of the statistical test are available in the third and fourth tabs of Supplementary Spreadsheet S1.

**Figure 4:**
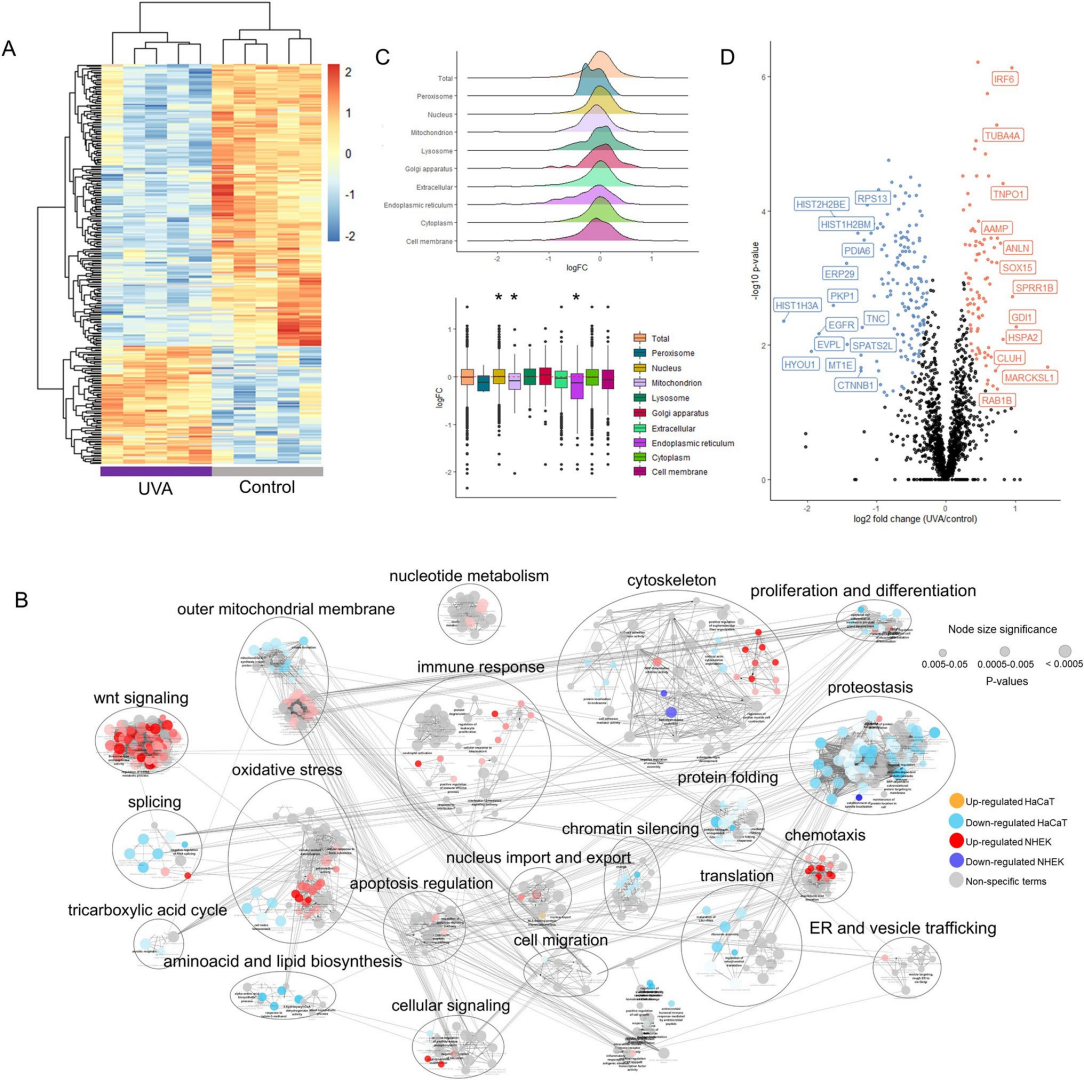
Proteome remodeling of HaCaT cells in response to UVA light. (**A**) Hierarchical clustering of differentially regulated proteins (Student’s T-test, 0.05 FDR correction) in HaCaT cells 24 h after exposure to UVA light. The color gradient represents z-scored LFQ intensities and columns represent replicates (n = 5 per group). (**B**) Enrichment network of the up and down-regulated proteins of HaCaT and NHEK cells post-exposure to UVA. Enriched GO-terms for biological processes are shown as nodes, which are interconnected according to the number of genes shared between them. The size of the nodes reflects the significance of the terms (*p*-value < 0.05, right-sided hypergeometric test, Benjamin-Hochberg FDR correction). Semantically related biological processes were clustered and labeled with AutoAnnotate to facilitate visualization of the network. (**C**) Compartment-specific proteome changes^27^ in irradiated versus control HaCaT cells 24 h after UVA exposure. Values represent log_2_(fold change UVA/control). (**D**) Volcano plot highlighting up and down-regulated proteins of irradiated HaCaT cells in relation to controls 24 h after UVA exposure. Up-regulated proteins are highlighted in orange and down-regulated are highlighted in blue. Significance was defined in this plot by a 0.05 FDR and labels were shown if log_2_(Fold Change) > 0.5 or < 0.5.

A Student’s T-test comparison, with 5% FDR correction, yielded 281 differentially abundant protein groups between control and irradiated HaCaT cells 24 h after treatment. The heatmap of differentially abundant proteins shows separation into two clusters, an up-regulated one, consisting of 83 proteins, and a down-regulated cluster of 198 proteins (Fig. 4A). HaCaT cells mostly down-regulates protein levels in response to the light stress, in opposition to NHEK cells that show a majoritarian cluster of up-regulated proteins, mostly involved in a potent response against UVA-induced stress.

To compare the effects of UVA on HaCaT and NHEK cells in the short-term, we generated a joint enrichment network considering significantly up and down-regulated proteins of NHEK cells, together with up and down-regulated proteins in HaCaT cells in response to UVA (Fig. 4B). The complete list of enriched biological processes of the joint network is available in the third tab of Supplementary Spreadsheet S2. The enrichment network shows that UVA radiation leads to a general reduction in anabolic processes as well as a clear pattern of up-regulation in catabolic proteins in HaCaT cells. For example, translation and semantically related terms are the most significantly enriched GO biological terms among down-regulated proteins. Ribosomal subunits and translation initiation factors and activators comprise 22% of all significantly down-regulated proteins in this cell type 24 h post-exposure to UVA. Epigenetic control of gene expression and RNA processing are also affected by light in immortalized cells (represented in the enrichment network by the term “chromatin silencing” (Fig. 4B)), with down-regulation of histone levels, i.e. subunits of histones H1, H2 and H3. Of note, core histone macro-H2A.1 is usually up-regulated in senescent cells, being required for SASP production^14^, and has been detected as up-regulated in NHEK cells exposed to UVA, in opposition to the down-regulation observed in HaCaT cells.

Another important differential regulation between the two cell types encompasses the processes “oxidative stress”, “chemotaxis”, “immune response” and “outer mitochondrial membrane” (Fig. 4B). Proteins represented by the “oxidative stress” process are mainly up-regulated in NHEK, which is in line with the senescent phenotype, but are down-regulated in HaCaT cells. Modulations in the levels of redox-responsive proteins by UVA in NHEK and HaCaT cells 24 h after irradiation possibly reflects the ability of each of the cell types to deal with reactive oxygen species (ROS) generated by UVA. It has been demonstrated that HaCaT cells display a fast nuclear translocation of Nrf2, a key regulator in the transcription of genes encoding antioxidant enzymes, in response to UVA light. Importantly, the same study shows that NHEK and HaCaT cells display contrary responses in most of the Nrf2-controlled proteins, which suggests that these cell types have different antioxidant capacities^36^. Similarly, proteins involved in immune activation are only up-regulated in NHEK cells 24 h after stress.

In opposition to the up-regulation of mitochondrial proteins observed in NHEK cells 24 h after exposure to UVA, all differentially expressed mitochondrial proteins (27 in total) are down-regulated in HaCaT (Fig. 4B). Besides down-regulation of respiratory chain components in HaCaT, a few enzymes from the citric acid cycle are also down-regulated. Accordingly, by analyzing changes in the proteome in a compartment-specific fashion, after 24 h we found a significant decrease in the fold changes of mitochondrial proteins of HaCaT cells in relation to the proteome (Wilcoxon test, 5% FDR correction) (Fig. 4C). These results are similar to those obtained with NHEK cells 7 days after irradiation, supporting the notion that HaCaT cells are more resilient to UVA-induced stress when compared to primary keratinocytes.

Even though the majority of differentially regulated proteins are down-regulated in HaCaT 24 h after irradiation, up-regulated proteins are specifically enriched for cytoskeleton reorganization (*MARCKSL1*, *SPRR1B*, *ANLN*, *TUBA4A*, *TUBA1C*, *MAPRE2*, *TUBB3*, *TTLL12*, *TUBA1B*, *EPPK1*). Cytoskeleton components and regulators of cytoskeleton organization are also among the proteins with the strongest statistical and practical significances between groups (e. g., *TUBA4A*, *ANLN*, *SPRR1B*, *MARCKSL1*) (Fig. 4D). *MARCKSL1*, the most up-regulated protein, is associated with increased migratory potential in cancer cells^37^.

### Senescent keratinocytes induce paracrine oxidative stress and immune system activation in pre-tumoral keratinocytes

The relationship between senescence and tumorigenesis has been described through the concept of antagonistic pleiotropy. Even though senescence is a mechanism of tumor suppression, senescent cells may secrete molecules that can stimulate tumorigenesis^24^. For example, senescent fibroblasts have been shown to stimulate proliferation of pre-malignant and malignant, but not normal, primary epithelial cells^38^. On the other hand, primary human keratinocytes have been shown to be susceptible to paracrine senescence^39,40^. Considering these evidences, we aimed at evaluating the paracrine effects of keratinocytes under UVA-induced senescence on the proteome of pre-tumoral, initiated HaCaT cells. HaCaT cells were used as a pre-tumoral model in this assay because they acquired p53 mutations during immortalization and have a defective senescence machinery. In this sense, HaCaT’s genetic background predispose these cells to malignancy and prevent these cells from entering paracrine senescence. Even though there are limitations to using an immortalized cell line as a pre-tumoral model, HaCaT’s acquired mutations in p53 can be useful in a pre-tumoral context because initiated, p53-mutated cells are commonly found in the skin after exposure to UV^41^.

**Figure 5:**
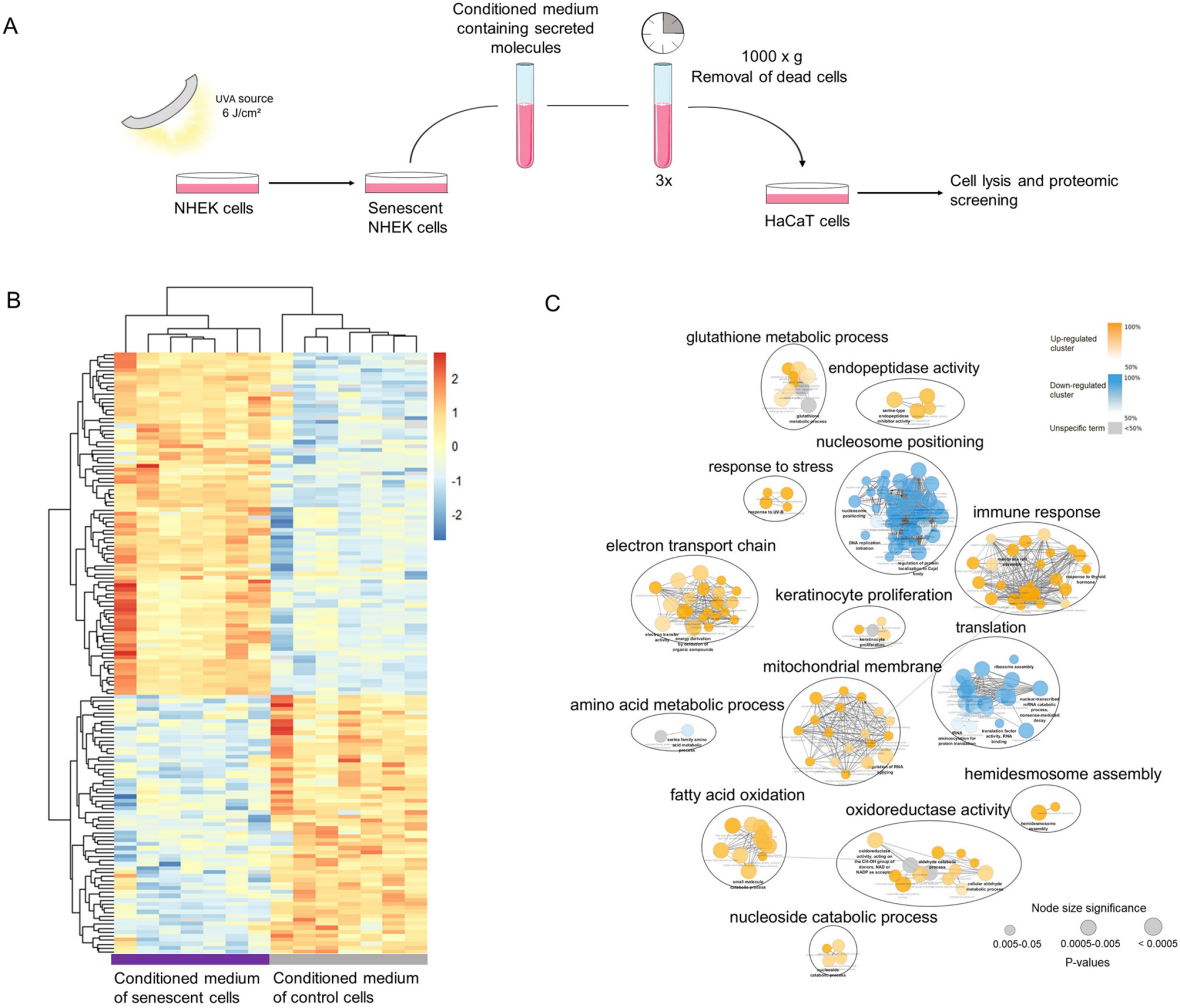
Proteome response of HaCaT cells to conditioned medium of senescent primary keratinocytes. (**A**) Scheme of the experimental approach performed to investigate bystander effects of senescent keratinocytes in immortalized cells. We acknowledge Servier Medical Art (https://smart.servier.com) for providing the illustrations of tubes. (**B**) Hierarchical clustering of differentially regulated proteins (Student’s t-test, 0.05 FDR correction) comparing HaCaT cells that received NHEK-irradiated secretome with those receiving the secretome of non-irradiated NHEK cells. The color gradient represents z-scored LFQ intensities and columns represent replicates (n = 5 per group). (**C**) Enrichment network of the up and down-regulated clusters identified in the heatmap showed in b. Enriched GO-terms for biological processes are shown as nodes, which are interconnected according to the number of genes shared between them. The size of the nodes reflects the significance of the terms (*p*-value < 0.05, right-sided hypergeometric test, Benjamin–Hochberg FDR correction). Related biological processes were clustered and labeled with AutoAnnotate.

For this assay, NHEK cells were irradiated with UVA light and maintained in culture for 7 days. On the first, third and fifth day after irradiation, conditioned medium containing NHEK-secreted molecules was centrifuged for removal of dead cells and transferred to HaCaT cells. In the seventh day, HaCaT cells were lysed and analyzed by mass spectrometry (a simplified scheme of the protocol is shown in Fig. 5A). In this experiment, control cells were treated with conditioned medium of non-irradiated primary cells.

Hierarchical clustering of 120 proteins with statistically different levels between HaCaT cells that received NHEK-irradiated secretome or the secretome of non-irradiated NHEK cells reveals the formation of up and down-regulated clusters, comprising 83 and 37 proteins, respectively (Fig. 5B). The label-free quantification of this dataset and the results of the statistical test are available in the fifth tab of the Supplementary Table S1. This result indicates the secretory phenotype of senescent keratinocytes is able to provoke changes in immortalized neighboring cells. The down-regulated cluster is mostly associated to translation, as has been previously observed to occur in UVA-exposed HaCaT cells (Fig. 5C). Interestingly, the up-regulated cluster is associated to immune system activation, detoxification of reactive species, and increased levels of structural mitochondrial proteins (Fig. 5C) (see Table 1 for specific proteins associated to these processes). These results suggest that exposure of immortalized HaCaT cells to senescent primary keratinocytes elicits a continuous anti-oxidant and pro-inflammatory response in these cells. Up-regulated proteins are also enriched for “keratinocyte proliferation”, suggesting modulations in the differentiation and proliferation capabilities of these cells. Immortalized keratinocytes also mimic some the responses of senescent keratinocytes by up-regulating laminin subunits (*LAMC2*, *LAMB3*, *LAMA3*) and few members of the serpin family (*SERPINB1* and *SERPINB13*), which are usually secreted in the SASP of senescent cells (Table 1). A complete list of enriched biological processes associated with the set of differentially abundant proteins is presented in the fourth tab of Supplementary Spreadsheet S2.

**Table 1:** Differentially regulated proteins in HaCaT cells treated with conditioned medium from senescent keratinocytes (proteins are associated with major significantly enriched GO-terms).

Process	Gene name	Protein name	Fold change
Immune system	LGALS7	Galectin-7	2.78
	S100A2	Protein S100-A2	2.78
	SERPINB1	Leukocyte elastase inhibitor	1.97
	SERPINB13	Serpin B13	1.87
	CAPG	Macrophage-capping protein	1.72
	LGALS3	Galectin-3	1.72
	SERPINB2	Plasminogen activator inhibitor 2	1.61
	CTSB	Cathepsin B	1.61
	SERPINB5	Serpin B5	1.58
	S100A14	Protein S100-A14	1.56
	S100A11	Protein S100-A11	1.52
	SERPINB8	Serpin B8	1.52
	S100A10	Protein S100-A10	1.39
	IRF6	Interferon regulatory factor 6	1.38
	S100A16	Protein S100-A16	1.32
Oxidative stress	PRDX5	Peroxiredoxin-5, mitochondrial	1.94
	GSTK1	Glutathione S-transferase kappa 1	1.51
	TXNDC17	Thioredoxin domain-containing protein 17	1.48
	AKR1A1	Alcohol dehydrogenase [NADP( +)]	1.43
	GSTP1	Glutathione S-transferase P	1.39
	GSR	Glutathione reductase, mitochondrial	0.75
Respiratory function	SQRDL	Sulfide:quinone oxidoreductase, mitochondrial	1.62
	COX6B1	Cytochrome c oxidase subunit 6B1	1.53
	SDHB	Succinate dehydrogenase [ubiquinone] iron-sulfur subunit	1.46
	NDUFA10	NADH dehydrogenase [ubiquinone] 1 alpha subcomplex subunit 10	1.39
Keratinocyte proliferation	IVL	Involucrin	4.19
	LAMA3	Laminin subunit alpha-3	2.37
	LAMC2	Laminin subunit gamma-2	2.37
	LAMB3	Laminin subunit beta-3	2.18
	CRABP2	Cellular retinoic acid-binding protein 2	2.00
	SERPINB13	Serpin B13	1.87
	PSAP	Prosaposin	1.86
	FERMT1	Fermitin family homolog 1	1.82
	ITG6A	Integrin alpha-6	1.60
	SRSF6	Serine/arginine-rich splicing factor 6	0.73
	CD109	CD109 antigen	0.68
	KRT18	Keratin, type I cytoskeletal 18	0.67

**Figure 6:**
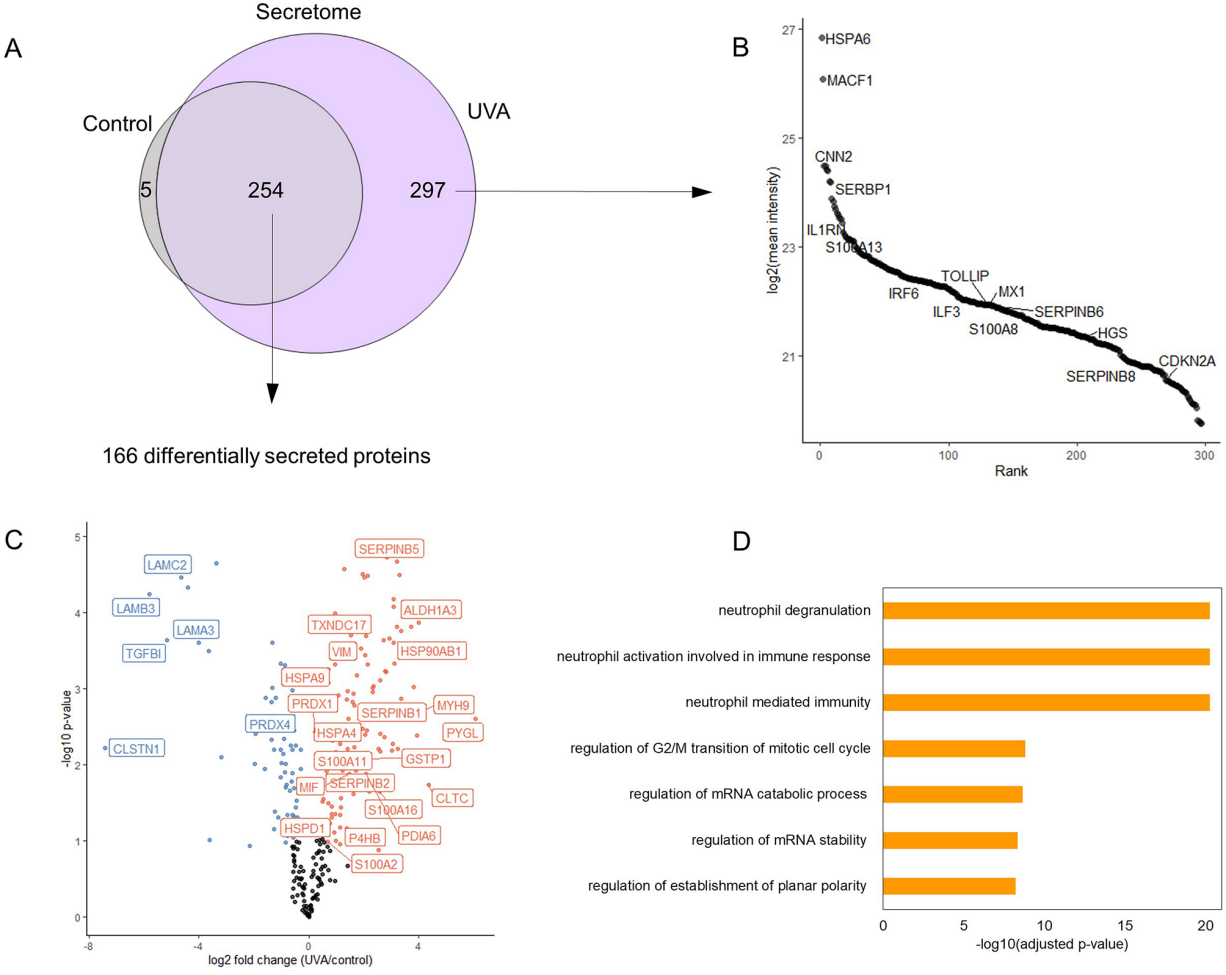
Secretome of NHEK cells irradiated with UVA light. (**A**) Venn diagram representing the secretome of control and irradiated cells (n = 3 per group). (**B**) Intensity-based ranking of proteins identified exclusively in the secretome of irradiated NHEK cells. (**C**) Volcano plot representing up and down-regulated proteins in the secretome of irradiated NHEK cells in comparison to controls 24 h after UVA exposure. Up-regulated proteins are highlighted in orange and down-regulated -proteins are highlighted in blue. Significance was defined in this plot by a 0.05 FDR and labels were shown if log_2_(Fold Change) > 0.5 or < 0.5. (**D**) Enrichment analysis of up-regulated proteins in the secretome of irradiated NHEK cells.

To investigate possible mediators of the paracrine responses in initiated HaCaT cells induced by keratinocytes under UVA-induced senescence, we analyzed the secretome of NHEK cells 24 h after exposure to UVA. A total of 254 proteins were quantified in all samples. Additionally, a total of 5 and 297 proteins were exclusively quantified in controls and UVA-irradiated samples, respectively (Fig. 6A). For a protein group to be considered present only in one condition, it had to be consistently quantified in all 3 biological replicates of this condition and completely absent in the other.

Consistently with our previous results, the levels of proteins involved in inflammatory processes were increased in the secretome of irradiated cells (Fig. 6B). Notably, p16 levels were also increased in irradiated cells compared to controls, consistently with the up-regulation we observed in cell lysates. The most enriched terms for up-regulated proteins in the secretome of irradiated cells are “neutrophil degranulation”, “neutrophil activation in immune response” and “neutrophil mediated immunity”, reinforcing the role of pro-inflammatory proteins in the paracrine effects triggered by cells under UVA-induced senescence in pre-tumoral keratinocytes.

For instance, we detected up-regulation in the secretion of a few members of the *SERPIN* and *S100* family (*S100A8, S100A13, S100A16, S100A2, SERPINB6, SERPINB8, SERPINB5, SERPINB1*), similarly to the trend we observed in lysates of cells exposed to UVA and conditioned medium (Fig. 6C–D). Cells under UVA-induced stress also differentially secrete *IL1RN*, *TOLLIP*, *MIF*, in agreement with the up-regulation observed for these proteins in lysates of NHEK cells 24 h after exposure to UVA. Furthermore, the levels of other relevant mediators of inflammation were altered in the secretome of irradiated cells, such as *IRF6, MX1, ILF3*. In this sense, *IRF6* and *MX1*, are involved in interferon signaling and are differentially regulated across most of the datasets we analyzed in this study. *MX1* is particularly up-regulated in NHEK cells 7 days post-exposure to UVA. *IRF6* is increased in HaCaT cells exposed to UVA and to the conditioned medium of senescent keratinocytes. *STAT1*, a transcription factor responsible for mediating type I interferon responses, is up-regulated in NHEK cells 24 h and 7 days post-exposure to UVA.

Additionally, the levels of redox-responsive enzymes were also increased in the secretome of irradiated cells compared to controls (*CAT*, *TXN* isoforms, *PRDX* isoforms, *PDI* isoforms, as well as a few *HSP*). It is possible that some of these proteins are secreted by cells under oxidative stress. It has been shown that cells under starvation, for example, increase ROS production and secrete antioxidant enzymes to prevent ROS-mediated damage in the extracellular space^42^. A complete list of up and down-regulated proteins in NHEK secretome is available in the first tab of Supplementary Spreadsheet S3. Additionally, the lists and label-free quantification data of proteins exclusively present in irradiated or control samples can be found in the second and third tabs of Supplementary Spreadsheet S3, respectively.

## Discussion

Skin aging is thought to be driven by intrinsic and extrinsic damage to biomolecules^15^. Cells burdened with accumulated damage may die and not be replaced, contributing to the functional decline of tissues^5^. On the other hand, cells may undergo phenotypic reprogramming and lose their proliferative capacity, a process that has been termed senescence^43^. In this context, UVA has been described to induce apoptosis in dermal fibroblasts, leading to dermal alterations usually observed during aging^5,44^. UVA has also been reported to induce senescence in skin fibroblasts, up-regulating p16, p21, p53, SA β-galactosidase, and *MMP1*^9,45^, thus playing a key role in dermal photoaging. Much less is known about the impact of UVA on the decline in epidermal function, even though epidermal photoaging is a risk factor for skin cancer and other skin diseases. While UVB-induced senescence has been well-characterized^46^, the only evidence to date suggesting senescence induction by UVA on keratinocytes relies on the fact that UVA modulates the levels of p63, ki67, and activated caspase‐3 in the germinative layer of the epidermis^47^. Importantly, senescence is a dynamic cellular reprogramming that may assume different phenotypes depending on the inducer and cell type^48^. Here, we characterized for the first time UVA-induced senescence in primary keratinocytes as dependent on anti-oxidant and pro-inflammatory responses, agreeing with UVA’s acknowledged oxidative action^3^. Senescence induction was confirmed by well-recognized senescence hallmarks (e.g., p16, SA β-galactosidase staining and impaired proliferation). In the short-term, up-regulation of structural mitochondrial proteins and alterations in translation components and regulators could reflect increases in mitochondrial mass and proteotoxic stress generally observed in stress-induced senescent phenotypes^17,21^. In the long-term, UVA-exposed cells still bear senescence markers, while exhibiting increased levels of lysosomal proteins and up-regulation of sequestosome-1, that could indicate the development of a late cellular repair response to cope with light stress. Senescent phenotypes have been shown to lead to ER stress, unfolded protein response and consequently autophagy as a mechanism for coping with proteotoxic stress^28^. UVA has also been reported to induce autophagic-lysosomal alterations that could result in impaired or late autophagic response^29^. On the other hand, immortalized keratinocytes are not as susceptible to the effects of direct UVA exposure as primary cells, recovering completely from UVA-stress in less than 7 days, probably due to adaptations developed by these cells during immortalization.

A few proteomics studies have evaluated the effects of UVA radiation on skin cells. In rats, chronic exposure to UVA modulates anti-oxidant proteins, inflammatory mechanisms and apoptosis in the skin^49^. In hairless mice, an acute dose of 20 J/cm^2^ of UVA light on the dermis affected mitochondrial function, calcium metabolism and cytoskeleton signaling. These effects were prevented by carnosine, a free radicals scavenger^50^. Exposure of hairless mice to 15 J/cm^2^ of UVA light lead to fibroblast senescent patterns characterized by accumulation of 4-hydroxynonenal (HNE) adducts, DNA damage and ubiquitinated proteins. Topical application of carnosine, prevented adducts formation, DNA damage and senescence induction^51^. In cultured fibroblasts, exposure to 20 J/cm^2^ of UVA light lead to changes in the levels of proteins associated with inflammation, anti-oxidant response and apoptosis. This study also revealed that rutin, an antioxidant with polyphenolic structure, protected fibroblasts from UVA-induced effects more efficiently than from UVB damage^52^. Moreover, Gęgotek and collaborators^53^ found that 20 J/cm^2^ of UVA up-regulates components of the DNA damage response, antioxidant proteins (peroxiredoxin-1, superoxide dismutase, glutathione reductase and glutathione S-transferase), as well as pro-apoptotic and inflammatory mechanisms in 3D cultured keratinocytes. Finally, another study^54^ based on a multi-omics approach revealed that 40 J/cm^2^ of UVA lead to the generation of phospholipid hydroperoxides, with consequent up-regulation of antioxidant enzymes (peroxiredoxin 6 and glutathione peroxidase) in primary keratinocytes. Importantly, both UVA exposure and administration of *in-vitro* UVA-oxidized phospholipids to keratinocytes lead to modulations in lipid metabolism and antioxidant mechanisms. Our work is in agreement with previous findings, reinforcing the role of oxidant and inflammatory mechanisms in the stress response of keratinocytes to UVA. In addition, our results show that stress response may accompany the development of a senescent phenotype in keratinocytes, and is relevant in an environmental context of exposure to a considerably low UVA dose of 6 J/cm^2^.

**Figure 7:**
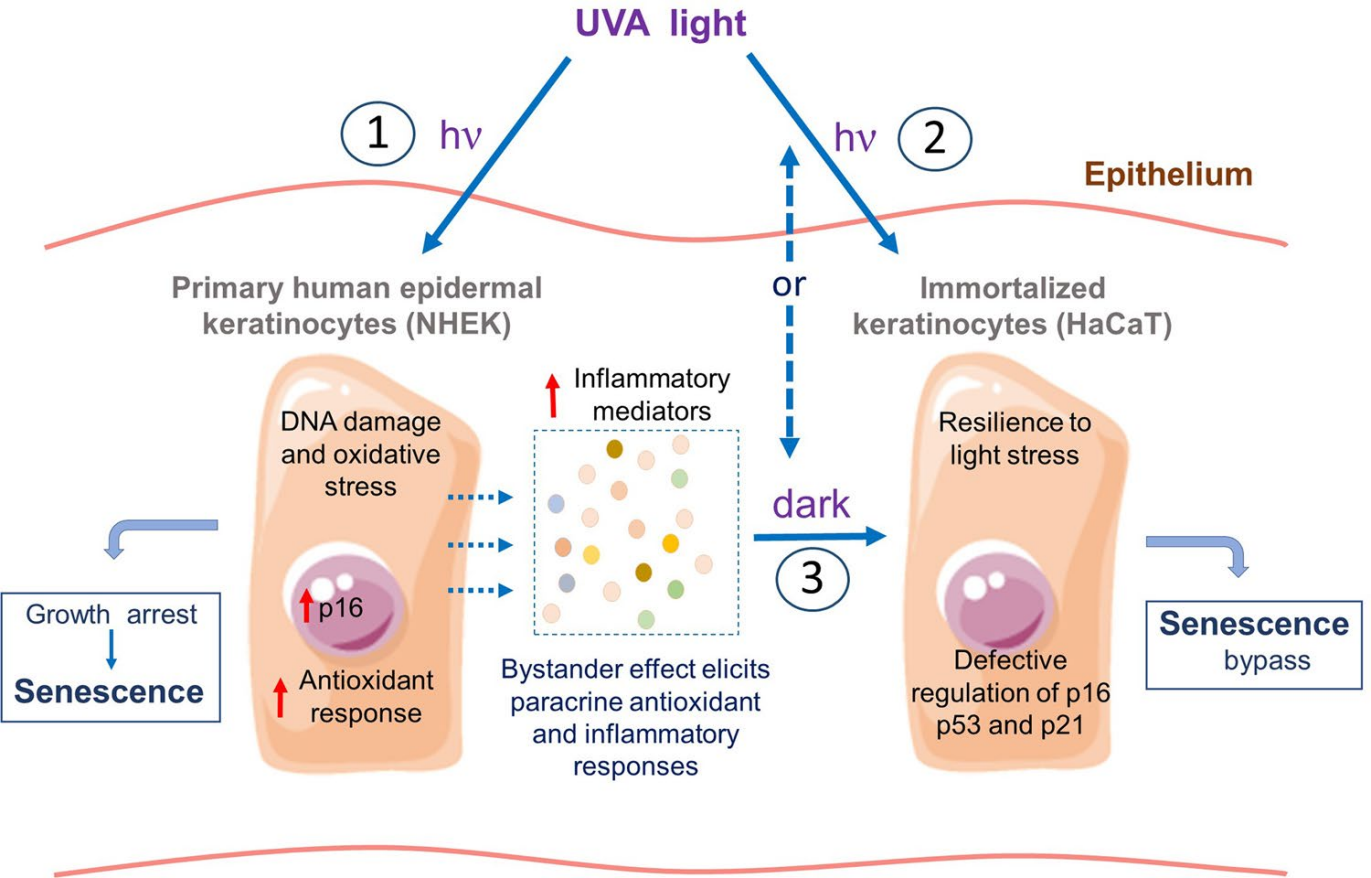
Proposed model of UVA-induced senescence in the epidermis. Genotoxic and oxidative stresses are biological effects of UVA light leading to the accumulation of senescent cells over time in the skin. Using cell-culture models, we show that UVA induces senescence in primary keratinocytes (**1**). Pre-malignant HaCaT cells are not as susceptible to the effects of direct UVA exposure as primary cells, probably due to adaptations developed by these cells during immortalization (**2**). However, HaCaT cells are susceptible to the effects of inflammatory mediators secreted by senescent keratinocytes (**3**). We acknowledge Servier Medical Art (https://smart.servier.com) for providing the illustrations of cells.

The control of the proliferative potential of keratinocytes in response to sun damage is at the heart of skin tumorigenesis, since all forms of skin cancer develop from epidermal cells and mainly from keratinocytes^55^. While senescent fibroblasts have been shown to stimulate the proliferation and malignancy of epithelial pre-tumoral cells, information about the paracrine effects of senescent cells on the proteome of pre-tumoral keratinocytes remains elusive. With that in mind, we characterized the response of pre-malignant HaCaT cells to neighboring senescent primary keratinocytes. HaCaT cells exposed to conditioned medium of senescent keratinocytes mimic the antioxidant and inflammatory responses developed during senescence, even though they do not suffer from permanent cell cycle arrest, as would be expected of primary cells susceptible to paracrine senescence^39,40^. Triggering of antioxidant response in HaCaT cells exposed to conditioned medium could reflect paracrine damage induction in HaCaT cells by senescent keratinocytes, as has been previously reported to occur in senescence phenotypes^40,56^. Secretome analysis revealed pro-inflammatory mediators are the main drivers of intercellular communication between primary and initiated cells. Importantly, phenotypic changes in both normal and initiated cells, accumulating oxidative damage and mutational load in combination with altered inflammation over time are factors contributing to how aging favors skin cancer^57^ (a simplified scheme of our proposed model is shown in Fig. 7).

## Methods

### Experimental model

HaCaT cells were kindly provided by Professor Mauricio S. Baptista (Institute of Chemistry, University of São Paulo) and were grown in DMEM basal medium supplemented with 10% fetal bovine serum, 100 U/mL penicillin and 100 ug/mL streptomycin. Normal human epidermal keratinocytes (NHEK) were obtained from Lonza (USA) and were cultured in Keratinocyte SFM basal medium supplemented with human recombinant epidermal growth factor and bovine pituitary extract (Thermo Fisher Scientific, USA). Cells were tested for mycoplasma contamination.

### Irradiation conditions

Irradiation was performed in an Oriel SOL-UV 2 solar simulator (Newport, USA) equipped with a Xenon arc lamp, an infrared bandpass filter and a UVB-blocking filter (the emission spectrum of the radiation emitted by the simulator is provided in Supplementary Fig. S1). Measurements of the output of the simulator were performed as described in^58^. The cells plates were positioned at a 10 cm distance from the light source and irradiated for 26 min to deliver a total dose of 6 J/cm^2^ of UVA light. In preliminary experiments, we tested two different doses of UVA radiation (6 and 12 J/cm^2^). Upon irradiation with 6 J/cm^2^, more than 90% of cells were viable^58^. Thus, we chose this dose to evaluate non-cytotoxic effects of UVA radiation on the proteome of skin cells.

After irradiation, cells were washed three times with Phosphate Buffer Saline (PBS), and kept in PBS during the irradiation. Control cells were kept in PBS and in the dark at room temperature during the whole irradiation period.

### Senescence associated β-galactosidase assay

Cells were irradiated and processed for the senescence-associated (SA) β-galactosidase assay^23^ seven days after treatment. Cells were washed two times with PBS, fixed for 4 min with 3% formaldehyde at room temperature, washed four times with PBS, and incubated at 37º C (in the absence of CO_2_) with the staining solution (1 mg/mL x-gal, 40 mM citric acid/Na phosphate buffer, 5 mM potassium ferrocyanide, 5 mM potassium ferricyanide, 150 mM sodium chloride, 2 mM magnesium chloride in water at pH 6), according to^23^. For each experiment and condition, 100 cells were counted.

### Proliferation assay

Cells were irradiated with UVA and processed 24 h and 7 days after exposure. For the proliferation assay^22^, cells were washed two times with PBS and incubated for 20 min at room temperature under agitation with 0.5% crystal violet. Cells were washed again 5 times with PBS, plates were scanned, crystals were resuspended in methanol (2 mL for each well of a 6-well plate) and the absorbance of each sample was measured. Measurements are accompanied by the trypan counting^59^ to assure that the assay assessed proliferation and not cell death.

### Secretome transfer experiment

NHEK cells were irradiated with UVA and maintained in culture for 7 days in order to develop a senescent phenotype. On the first, third and fifth day after irradiation, the conditioned medium containing secreted molecules was centrifuged three times for 15 min at 1000 × g for removal of dead cells and transferred to HaCaT cells. On the seventh day, HaCaT cells were washed 5 times with PBS, lysed and proteins were analyzed by mass spectrometry. Control cells were treated with the conditioned medium of non-irradiated primary cells.

### Secretome sample preparation

NHEK cells were irradiated in PBS and kept overnight in DMEM basal medium, supplemented with KGM™ Gold Keratinocyte Growth Medium SingleQuots™ Supplements and Growth Factors (Lonza), without fetal bovine serum and phenol red. The supernatants were collected and centrifuged 3 times for 15 min at 3000 × g. Afterwards, the samples were concentrated in 3 kDa Amicon ® Ultra cut-off filters (Millipore). Final protein concentration was measured using a Pierce™ BCA Protein Assay Kit (Thermo Fisher Scientific), according to the manufacturer’s instructions.

### Cell lysate sample preparation

For proteomics experiments, 400,000 cells were plated in 6-well plates 24 h before the experiments. After irradiation, cells were washed five times with PBS, and scraped in 500 µL of a solution containing 100 mM ammonium bicarbonate, 8 M urea and protease inhibitors (cOmplete™ Protease Inhibitor Cocktail, Roche). Cell lysates were kept on ice for one hour and, after that, they were precipitated overnight with 3 volumes of cold (-20 °C) acetone. Precipitated proteins were collected by centrifugation. Pellets were air-dried for about 10 min and resuspended in 100 mM ammonium bicarbonate buffer containing 8 M urea. Protein concentration was measured using a Pierce™ BCA Protein Assay Kit (Thermo Fisher Scientific).

### Protein digestion

A total of 10 µg of protein per sample was reduced, alkylated and digested. Reduction was performed with 5 mM of dithiothreitol for 1 h (at 30 °C) and alkylation with 15 mM of iodoacetamide for 30 min (at 30 °C), under agitation (400 rpm). Before digestion, proteins in 8 M urea were diluted 10 times with ammonium bicarbonate. Digestion was performed overnight by addition of two aliquots of trypsin (in the proportions of 1:40 and 1:50 w/w of trypsin:protein, respectively, with an interval of 4 h between the first and second additions), under agitation (400 rpm), at 30 °C. Digestion was stopped by using 4% trifluoroacetic acid. Afterwards, samples were dried and desalted using the StageTip protocol described by^60^. Peptides were washed 10 times in the StageTips^60^ with 0.1% TFA and subsequently eluted in 50% acetonitrile, 0.1% TFA. This protocol has been previously described in^58^.

### LC–MS/MS measurements

The following LC–MS/MS analysis was performed as previously described in^58^. Each sample was analyzed in an Orbitrap Fusion Lumos mass spectrometer coupled to a Nano EASY-nLC 1200 (Thermo Fisher Scientific, Bremen, Germany). Peptides were injected into a trap column (nanoViper C18, 3 μm, 75 μm × 2 cm, Thermo Scientific) with 12 µL of solvent A (0.1% formic acid) at 980 bar. After this period, the trapped peptides were eluted onto a C18 column (nanoViper C18, 2 μm, 75 μm × 15 cm, Thermo Scientific) at a flow rate of 300 nL/min and separated with a gradient of 5–28% acetonitrile with 0.1% formic acid for 80 min, followed by 28–40% acetonitrile with formic acid for 10 min. The eluting peptides were detected in data-dependent acquisition mode using positive electrospray ionization. A full scan (m/z 400–1600) was recorded at a 60,000 resolution, followed by HCD fragmentation of the most intense ions, considering an intensity threshold of 5 × 10^4^. Ions were filtered for fragmentation by a quadrupole with a transmission window of 1.2 m*/z*. HCD fragmentation was performed with a normalized collision energy of 30 and the fragments were analyzed by the Orbitrap at resolution of 30,000. The number of MS2 events between full scans were determined by a cycle time of 3 s. A total of 5 × 10^5^ and 5 × 10^4^ ions were injected in the Orbitrap with an accumulation time of 50 and 54 s for the full scan and MS2 acquisition, respectively. Monocharged ions or ions with undetermined charge were not selected for fragmentation.

### Proteomics data analysis

Raw files were processed using MaxQuant^61^. The Andromeda algorithm^62^ was used for protein identification against the homo sapiens Uniprot database (downloaded August, 2019; 20,416 entries). Error mass tolerance for precursors and fragments were set to 4,5 ppm and 0,5 Da, respectively. Cysteine carbamidomethylation was selected as a fixed modification and methionine oxidation and *N*-terminal acetylation were selected as variable modification. Trypsin was set as digestion enzyme, with a maximum of 2 missed cleavages allowed. A maximum FDR of 1% was allowed both for peptides and proteins identification, and for proteins it was calculated using a decoy database created from the reverse ordination of the protein sequences in the Uniprot database. Identification of at least two peptides (unique + razor) was set as a parameter for the identification of a protein. Protein abundances were quantified by the LFQ algorithm, based on the normalized chromatographic peak integrations generated by MaxQuant. Other parameters were kept as default.

Before statistical analysis, the data were log-transformed, and matches to the contaminants and reverse database, as well as proteins identified only by modified sites, and missing values (< 30% in all samples) were filtered out. Statistical significance was assessed using a two-tailed Student’s T-test in the Perseus software^63^ with a permutation-based false discovery rate (FDR) of 5% and a S_0_ parameter of 0.1. The plots were displayed in the R statistical computing environment, using standard libraries, ggplot2 and pheatmap. The number of independent replicates for each experiment is specified in the legends of their respective figures.

### Network analysis

For functional network analysis, we used the ClueGO^12^ and AutoAnnotate^64^ apps in Cytoscape^65^. ClueGO presents enrichment analysis as a network of interconnected nodes representing gene ontology (GO) biological processes, with edges representing kappa scores (a statistic based on the number of genes shared between different biological processes)^12^. Benjamin-Hochberg correction for multiple hypotheses was applied to the results of a hypergeometric test based on the enrichment analysis and all networks represent processes that have a corrected *p*-value lower than 0.05. Clusterization of semantically-related terms was performed with AutoAnnotate to facilitate visualization of the network. The output tables of ClueGO enrichment analysis are provided in Supplementary Table S2.

## Supplementary Information

Supplementary Information 1.

Supplementary Information 2.

Supplementary Information 3.

Supplementary Information 4.

## Data Availability

The proteomic datasets generated during this study have been deposited to the ProteomeXchange Consortium (http://www.proteomexchange.org/) via the PRIDE repository (identifier: PXD025191).
