# Supplementary material for: A single dose of Ultraviolet-A induces proteome remodeling and senescence in primary human keratinocytes
Source: Sci Rep. 2021 Dec 2;11:23355. doi: 10.1038/s41598-021-02658-5 (PMC8639817; doi:10.1038/s41598-021-02658-5)
Supplement: Supplementary file 4 — Supplementary Information 4. [file 41598_2021_2658_MOESM4_ESM.pdf]

Supplementary Figure

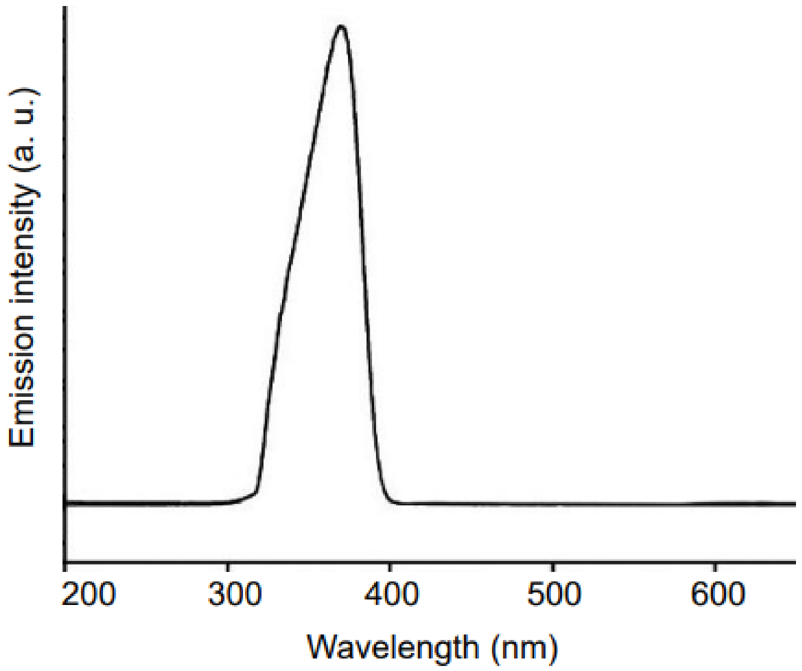

Figure 1S: Emission spectrum of the UVA radiation emitted by the Oriel SOL-UV 2 solar simulator.

Supplementary spreadsheets:

Spreadsheet S1 - Protein-level Perseus data table output for the expression datasets

This spreadsheet contains the output of the Perseus software for the datasets involving changes in protein expression: label-free quantification data, protein identification information and statistical significance of altered proteins. Each tab refers to a particular dataset: NHEK cells 24 hours and 7 days post-irradiation (tabs 1 and 2, respectively), HaCaT cells 24 hours and 7 days post-irradiation (tabs 3 and 4, respectively) and HaCaT cells exposed to the conditioned medium of senescent keratinocytes (tab 5).

Spreadsheet S2 - ClueGo enrichment data output

This spreadsheet contains the table outputs of the ClueGo enrichment analysis represented in Figures 1, 4 and 5 (tabs 1, 2 and 3, respectively).

Spreadsheet S3 - Protein-level data table for the secretome dataset

The first tab of this spreadsheet contains the label-free quantification data, protein identification information and statistical significance of altered proteins for the dataset involving changes in protein secretion by primary human keratinocytes 24 hours post-exposure to UVA (6 J/cm^2). Tabs 2 and 3 include label-free quantification data of proteins quantified exclusively in one condition (control or UVA-irradiated) of this same experiment.
